# Editorial: Wildlife parasitology: emerging diseases and neglected parasites

**DOI:** 10.3389/fvets.2024.1439564

**Published:** 2024-07-03

**Authors:** Georgiana Deak, Nina Germitsch, Alicia Rojas, Alireza Sazmand

**Affiliations:** ^1^Department of Parasitology and Parasitic Diseases, University of Agricultural Sciences and Veterinary Medicine of Cluj-Napoca, Cluj-Napoca, Romania; ^2^Pathology and Microbiology, Atlantic Veterinary College, University of Prince Edward Island, Charlottetown, PE, Canada; ^3^Laboratory of Helminthology, Faculty of Microbiology, University of Costa Rica, San José, Costa Rica; ^4^Department of Pathobiology, Faculty of Veterinary Medicine, Bu-Ali Sina University, Hamadan, Iran

**Keywords:** wildlife, parasites, international, neglected, pathogens

## Introduction

The scientific literature involving wildlife and parasites has been mainly focused on the most common carnivore species, with the majority of publications originating in Europe. The present Research Topic was edited by parasitologists from different geographical areas (Romania, Canada, Costa Rica, and Iran), who managed to gather an impressive number of papers focused on wildlife parasitology from various countries, contributing to the extension of the knowledge in this field as well as highlighting the importance of research in this research area.

Overall, 15 original research papers and four case reports were included in this Research Topic, published by 147 different authors based in 25 countries. Among these, 3 (15.8%) papers were focused on carnivore species, 1 (5.3%) investigated parasites in primates, 3 (15.8%) papers reported parasites of wild birds, 1 (5.3%) on armadillos, 2 (10.5%) on wild boars, 1 (5.3%) on bats, 1 (5.3%) on invasive frogs, 1 (5.3%) on marsupials, and 4 (21%) investigated parasites in ruminants. In addition, one paper (5.3%) was focused on cats and one (5.3%) on fleas. Of note in this Research Topic two novel parasite species namely *Sarcocystis funereus* (Apicomplexa, Sarcocystidae), and *Delicata tatouay* (Molineidae, Anoplostrongylinae) were described, and the existence of the ancient deer-specific *Cooperia ventricose* was confirmed.

Bellow we summarize 19 articles sorted by their subject to three classical categories i.e., helminths, protozoa, and arthropoda.

## Articles on helminth parasites

Wild canids are known as important reservoirs of zoonotic parasitic infections. Uribe et al. investigated the presence of zoonotic helminths in wild canids from the Amazonian and Andean regions in Colombia and identified three species with a zoonotic potential (*Dipylidium caninum, Spirometra mansoni*, and *Lagochilascaris cf. minor*). A better knowledge of epidemiology and transmission routes of these neglected helminths is advocated.

Among wild carnivores, mustelids represent a less studied group, although very abundant and widely distributed. Deak et al. conducted a study on the species diversity and distribution of *Crenosoma* species infecting mustelids in Romania and showed that badgers were infected by *C. melesi* and *C. petrowi*, while beech martens were infected with *C. petrowi* and *C. vulpis*. The authors reported new host-parasite associations and sequenced *C. melesi* and *C. petrowi* for the first time.

Zoonotic parasitic infections are very diverse and can also be caused by metastrongyloid nematodes. One example of such a parasite infecting an atypical host is presented by Solorzano-Scott et al. in an opossum from Costa Rica. The authors presented the first case of cerebral infection by *Angiostrongylus costaricensis*, underlying the difficulties in diagnosing neuroangiostrongylosis and the importance of molecular methods to confirm the identity of parasites.

Felids are important hosts for many parasites, some of which have a severe or even lethal effect. Such parasites, like the neglected angio-neurotropic *Gurltia paralysans* nematode, can cause severe disease in domestic cats and can pose a risk for endangered wild felids. Feline gurltiosis cases from South America were presented by Gómez et al. in an original research paper with a focus on a specific case of infection in a domestic cat.

Non-human primates are commonly observed in captivity. Cuccato et al. reported a lethal case *of Cysticercus longicollis* infection in a captive ring-tailed lemur (*Lemur catta*) from Italy. The exact source of infection was not identified, but it was assumed to be correlated with carnivores from the biopark, underling the importance of the control of parasitic diseases as well as the implementation of biosecurity measures.

Magdálek et al. studied the seasonality and anthelmintics susceptibility of *Ashworthius sidemi*, an alien nematode which has emerged in captive fallow deer in Central and Eastern Europe over the last decade. Negligible seasonal patterns of parasite egg shedding indicated adaptation of this non-native parasite to the current climatic conditions of the Czech Republic.

Albrechtová et al. revised the trichostrongylid nematode *Cooperia* from red deer (*Cervus elaphus*) and sika deer (*Cervus nippon*) and confirmed the existence of the deer-specific *Cooperia* species *C. ventricose* which was described only in 1809 and is similar in morphology to *C. pectinata* parasitizing bovids.

Pikula et al. reported the filarial nematode *Litomosa* sp. in the abdominal cavity of a parti-colored bat (*Vespertilio murinus*) and its microfilariae in bat semen suggesting semen-borne transmission of this worm in addition to the known life cycle pattern that involves blood-sucking ectoparasites.

de Oliveira Simões et al. described a new roundworm *Delicata tatouay* in the small intestine of the greater naked-tailed armadillo (*Cabassous tatouay*) that inhabits Uruguay, Northeastern Argentina, Eastern Paraguay, and South, Central, and Northeastern Brazil. This novel species is the 14th member of the genus *Delicata* that infects armadillos.

Finally, Lykins et al. reported encysted larvae *Pterygodermatites whartoni* in invasive Cuban treefrogs (*Osteopilus septentrionalis*) in Central Florida, United States. Authors demonstrated that Cuban treefrogs can serve as potential paratenic hosts of *P. whartoni*, that the spirurid nematode is not restricted to Southeastern Asia, and that this invasive frog can play a role in the distribution and transmission of the invasive parasite.

## Articles on protozoan parasites

Wild boars have a wide geographical distribution and are susceptible to many parasitic infections, representing an important reservoir for pathogen transmission to animals and humans. In Europe, the population of wild boars has increased, and as a consequence, the risk of emerging vector-borne diseases is higher. Sgroi et al. screened over 200 wild boars from Italy for *Babesia*/*Theileria* infections and identified for the first time *Babesia vulpes* and *Babesia capreoli* in 13 and 2 tested wild boars, respectively. In the same animal species, but this time in Asia (Korea), Lee and Kwak explored the public risk of infection with *Giardia duodenalis* by examining 612 wild boar fecal samples using the PCR technique. Overall, they identified 20.4% prevalence in wild boars underlying the seasonal factor as an important risk factor.

The pudus (*Pudu puda*) are the world's smallest deers, and distributed only in the Southern Andes of Chile and Argentina. Hidalgo-Hermoso et al. reported DNA of *Bartonella* spp., hemotropic *Mycoplasma ovis*-like, and *Coxiella burnetii* from this near threatened Cervidae all of which are potentially zoonotic. They presented the first report of *B. henselae*, the causative agent of zoonotic cat scratch diseases, in a wild ungulate.

Máca et al. identified the Tengmalm's owl (*Aegolius funereus*) as the definitive host of a novel *Sarcocystis* species named as “*Sarcocystis funereus* sp. nov.” Authors isolated oocysts and sporocysts of the parasite from the intestinal mucosa of the bird host, and experimentally fed them to a mouse model (as intermediate host) to observe the sarcocysts in the skeletal muscle.

## Articles on arthropod parasites

Bahiraei et al. reported 31 chewing lice species collected from 612 examined wild birds representing 16 orders, 33 families, 60 genera, and 78 species in different regions of Iran. They also presented an updated checklist of louse species reported from the country according to their avian hosts.

Liu et al. worked on the mitochondrial genomes of two flea species *Frontopsylla spadix* and *Neopsylla specialis* which are the main flea vectors for the transmission of wild rodent plague into rats. Data generated using long-range PCR and next-generation sequencing technologies made the basis for future molecular evolution, taxonomy, and systematics of the flea species.

Sarcoptic mange in the Spanish wild goat Iberian ibex (*Capra pyrenaica*) was the subject of a study by Valldeperes et al.. They found that the local skin immune response is a determinant factor in the clinical responses to *Sarcoptes scabiei* infestation in this species.

Wechtaisong et al. studied the diversity of *Anaplasma* and *Bartonella* species in *Lipoptena fortisetosa* keds collected from captive Eld's deer (*Rucervus eldii thamin*) in Thailand. Authors discovered five *Bartonella* lineages including a new independent lineage of novel *Bartonella* species, *Anaplasma bovis* and other ruminant-related *Anaplasma*. They suggested the implementation of preventative measures in areas surrounding wild animals in order to prevent pathogen transmission among animals and humans.

Ali et al. reported for the first time the soft tick *Alectorobius coniceps* collected from nests of highly aerial birds “swifts” in Pakistan with morphological and genetic data expanding its reported geographical distribution.

## Conclusion

In conclusion, this Research Topic successfully addressed a variety of parasitic groups in a myriad of hosts from different regions of the world. This richness demonstrates the fertile ground wildlife parasitologists are working on and the importance of One Health approaches to keep this important research.

## Author contributions

GD: Conceptualization, Writing – original draft. NG: Writing – review & editing. AR: Writing – review & editing. AS: Writing – original draft.

